# Collapsible Soil Stabilization Using a Novel Crosslinked Biopolymer Binder: A Multiscale Evaluation

**DOI:** 10.3390/polym18192376

**Published:** 2026-09-29

**Authors:** Hadi Fatehi, Delaram Bahrampour, Mahsa Salehi Nia, Alireza Fatehi, Ilhan Chang

**Affiliations:** 1 School of Engineering and Built Environment, Griffith University, Nathan, QLD 4111, Australia; 2 Department of Civil Engineering, Shahid Rajaee Teacher Training University, Tehran 16788-15811, Iran; 3 Department of Civil & Environmental Engineering, K. N. Toosi University of Technology, Tehran 19967-15433, Iran; 4 School of Engineering, Shahid Bahonar University of Kerman, Kerman 76169-13439, Iran; 5 Department of Civil System Engineering, Ajou University, Suwon 16499, Republic of Korea; ilhanchang@ajou.ac.kr

**Keywords:** sustainable soil stabilization, collapsible soils, soy protein isolate, crosslinked polymer network, bio-based stabilizer, biopolymer stabilization

## Abstract

In this study, soy protein isolate, a renewable protein-based biopolymer containing a minor carbohydrate fraction, was formulated into a polymeric binder using polyvinyl alcohol (PVA) and oxidized glucose and applied to improve the hydro-mechanical behavior of collapsible soils. The resulting soy protein–PVA-based binder (CSP) was evaluated against chitosan through laboratory tests including collapse potential, unconfined compressive strength (UCS), consolidated drained triaxial testing, permeability, and microstructural characterization using scanning electron microscopy (SEM) and Fourier transform infrared spectroscopy (FTIR). CSP substantially improved the engineering performance of the treated soils. For Soil C, the collapse potential decreased from 9.3% for the untreated soil to 3.0% with only 0.25% CSP. At 1.5% CSP and 9 days of curing, the UCS reached 2367 kPa for Soil C. In consolidated drained triaxial testing, the cohesion of soil C increased from 5 kPa in untreated soil to 734 kPa with 1% CSP, while the corresponding friction angle increased from 24° to 28°. FTIR and SEM observations indicated changes in the chemical environment and composite microstructure after treatment, although FTIR alone does not establish covalent crosslinking. Overall, the results demonstrate the potential of the CSP-based binder to improve the mechanical and collapse behavior of collapsible soils under the investigated laboratory conditions.

## 1. Introduction

Problematic soils present significant challenges in geotechnical engineering and its applications. Collapsible soils are problematic because their volume, strength, and mechanical properties are strongly influenced by moisture content [1]. This phenomenon usually occurs because of high porosity, low unit weight, weak particle bonding, and an unstable structure. These soils are mainly fine-grained, and particle bonding is established by cementing agents such as iron oxide, calcium carbonate, or by capillary forces [2]. Changes in moisture content can weaken these bonds, reducing strength and causing unwanted settlement in structures. As a result, understanding the mechanical behavior of these soils and finding effective solutions to enhance their geotechnical properties are crucial.

The geotechnical behavior of collapsible soils can be improved using conventional methods such as dynamic compaction, water injection, and stabilizers like cement and lime, as well as sustainable approaches such as microbial-induced calcite precipitation (MICP), nanomaterials, and biopolymers. The selection of a suitable method depends on soil conditions, environmental factors, and project requirements [3,4]. Because of the negative impacts of conventional materials (lime and cement), sustainable methods have attracted more attention in recent decades. Biopolymers, known for their environmental friendliness, have proven effective for soil improvement. Previous studies have indicated that biopolymers can enhance geotechnical properties by increasing shear and compressive strength, reducing liquefaction potential, and controlling erosion [5]. Biopolymers, with their high specific surface area, varied electrical charges, and diverse particle sizes, interact with fine particles (silt and clay) via ionic and hydrogen bonds while also enhancing the mechanical properties of sand. These interactions yield a more stable and improved soil structure [6,7,8,9,10,11,12].

As mentioned above, collapsible soils exhibit rapid and severe volume change upon saturation, which results in lower shear strength, high settlement, and structural instability. Biopolymers have already demonstrated their potential to address the challenges of collapsible soils, offering a sustainable solution due to their biodegradability and renewable nature [1,13,14,15,16,17,18,19,20]. Their utilization reduces reliance on chemical additives and promotes ecosystem health [21]. Previous research indicates that biopolymers such as sodium alginate, xanthan gum, and guar gum effectively improve soil properties. For instance, a 3.0% concentration of sodium alginate significantly improved the UCS and California bearing ratio of alluvial soil [22]. In another study, xanthan gum outperformed guar gum in stabilizing black cotton soil. Additionally, increasing the biopolymer concentration enhanced the maximum dry density and reduced the optimum moisture content [23]. Separately, the combination of xanthan gum with geosynthetics improved shear strength and cohesion [24,25]. The UCS test results indicate that adding chitosan improves soil strength over time, with an optimal curing temperature between 45 and 65 °C. SEM analysis confirms that chitosan effectively bonds soil particles, enhancing strength and stability [16].

Recent studies have further demonstrated the effectiveness of chitosan-based stabilization for fine-grained soils under both soaked and unsoaked conditions, with improvements maintained over extended curing periods [26]. These findings highlight the potential of chitosan for applications where long-term mechanical performance and water resistance are required. However, the development of alternative bio-based binder systems remains necessary to explore sustainable and potentially more adaptable stabilization approaches. In this regard, the present study investigates a crosslinked soy protein–PVA–oxidized glucose (CSP) system and compares its performance with chitosan to evaluate its potential as an alternative biopolymer-based stabilizer [27,28,29].

Similarly, polyvinyl alcohol (PVA) enhances soil properties, improving expansive soil strength with peak shear strength at 3% PVA. In alkaline soils, PVA increases aggregate stability, and cohesion (0.75–1.25%) increases strength by 43.5% and 34.9%, respectively, while xanthan gum and sodium alginate (4%) reduce the collapse index by up to 96% and enhance shear strength by 145% and 106%, respectively [8,30]. However, these studies address only specific aspects of collapsible soil behavior, leaving many challenges unexamined. Additionally, the long-term durability and environmental impact of synthetic polymers require further investigation.

This research investigates stabilizing collapsible soils with crosslinked soy protein–polyvinyl alcohol (CSP) as the main treatment, using chitosan as a comparison benchmark because of its well-known water resistance. Although soil improvement based on biopolymers has received attention, the specific problem of reducing collapse potential, especially under saturated conditions, has not been adequately addressed in the literature. Most of the existing studies have focused on strength improvement, but the collapse behavior, which is important for the stability of unsaturated soils, has been neglected. To bridge this gap, the present study introduces a novel CSP formulation consisting of soy protein, the synthetic polymer polyvinyl alcohol (PVA), and oxidized glucose as a bio-based crosslinking agent, with the aim of enhancing the water resistance and structural integrity of soy protein for soil stabilization. Chitosan was included to compare its performance with CSP and improve the comparative assessment. The study includes laboratory experiments to assess in depth the effects of these treatments, such as collapse potential, mechanical strength, permeability, and microstructural analyses. The present study offers new perspectives on environmentally sustainable ground improvement approaches for practical geotechnical applications, exploring the interaction between biopolymers and the behavior of collapsible soil.

## 2. Materials and Methods

### 2.1. Crosslinked Soy Protein & Polyvinyl Alcohol (PVA)

Soy protein isolate was purchased from Sigma-Aldrich (Product No. S0887, CAS No. 9010-10-0, Melbourne, Australia). According to the supplier, it contains approximately 90% protein, 5–6% carbohydrates, and less than 1% fat. The carbohydrate fraction consists mainly of oligosaccharides and polysaccharides with molecular weights in the range of 1–10 kDa. Soy protein is a water-insoluble, globular protein primarily composed of glycinin (11S) and β-conglycinin (7S) subunits.

Polyvinyl alcohol (PVA) was purchased from Sigma-Aldrich (CAS No. 9002-89-5, Australia) with a molecular weight range of 85,000–124,000 g/mol and a degree of hydrolysis of approximately 87–89%. This corresponds to a partially hydrolyzed PVA grade that is suitable for applications requiring moderate water solubility and good film-forming characteristics. The material was used as received without further purification.

This study used oxidized glucose as a bio-based crosslinking agent. The aldehyde groups generated by controlled oxidation of D-(+)-glucose (Sigma-Aldrich, ≥99% purity, Product No. G7528, Australia) reacted with amine groups in soy protein and hydroxyl groups in PVA.

### 2.2. Chitosan

Chitosan was purchased from Chemsupply (CAS No. 9012-76-4, Gillman, Australia). It had an average molecular weight of 120–140 kDa and a degree of acetylation (DA) of approximately 15%. No further modifications were made before use.

### 2.3. Soils: Sand and Kaolinite

The sand used in this study was collected from a construction site located on the Gold Coast, Australia. The mineralogical composition of the sand was determined using X-ray diffraction (XRD) and is presented in Table 1. Geotechnical parameters are summarized in Table 2.

Kaolinite clay with a known mineralogical composition was used in the experiments. Its chemical and geotechnical characteristics are presented in Table 3 and Table 4. The chemical composition of the kaolinite was determined using X-ray fluorescence (XRF) analysis. The clay had a specific gravity of 2.56, a plastic limit of 24.24%, and a liquid limit of 31.3%. According to the ASTM soil classification system, it is categorized as low-plasticity clay (CL), with a pH of 4.5.

## 3. Research Method

### 3.1. Collapsible Soil Preparation

In this study, laboratory-prepared collapsible soil was employed, created by mixing kaolin clay with sand at a specific moisture content. Collapse potential (CP) is defined as the ratio of the collapse settlement (ΔH) to the original thickness of the soil sample (H_0_), as shown in the following formula:

CP = ΔH/H_0_

where ΔH is the collapse settlement, and H_0_ is the original thickness of the soil sample.

Details of the kaolin clay used in this project are presented in Table 3 and Table 4. The laboratory-prepared collapsible soils were prepared following the specimen-preparation approach reported by Hanna and Soliman [33]. The sand and kaolin were thoroughly mixed at an initial moisture content of 5%. A 100-g hammer with a drop height of 20 cm was then used to apply 10 blows to the soil within the specimen mold. In this study, the number of blows was treated as a controlled specimen-preparation parameter rather than as a standard compaction criterion. The purpose of this procedure was to reproducibly generate a metastable soil fabric representative of collapsible soils. The same preparation procedure was applied to all soil compositions to ensure consistency among specimens. The resulting unit weights of Soil A, Soil B, and Soil C were 16.33, 16.25, and 16.08 kN/m^3^, respectively (Table 5).

In the final experimental setup, three samples with clay contents of 10%, 15%, and 20% were used. For these samples, an initial moisture content of 5% and a compaction energy of 10 blows with a 100-g hammer dropped from a height of 20 cm were applied. The measured collapse potentials for these samples were 5.76%, 7.20%, and 9.35%, respectively. Further details on these samples are provided in Table 5. The procedure for the preparation of the laboratory-prepared collapsible soil was adopted from established methodologies reported in previous geotechnical studies. The chosen ratios of sand and kaolin and the controlled initial moisture content and compaction energy were selected to mimic the metastable fabric and collapse behavior of natural collapsible soils. These preparation methods are widely used in the literature and are considered reliable and reproducible methods for the laboratory study of collapsible soil behavior [33].

**Figure 1:**
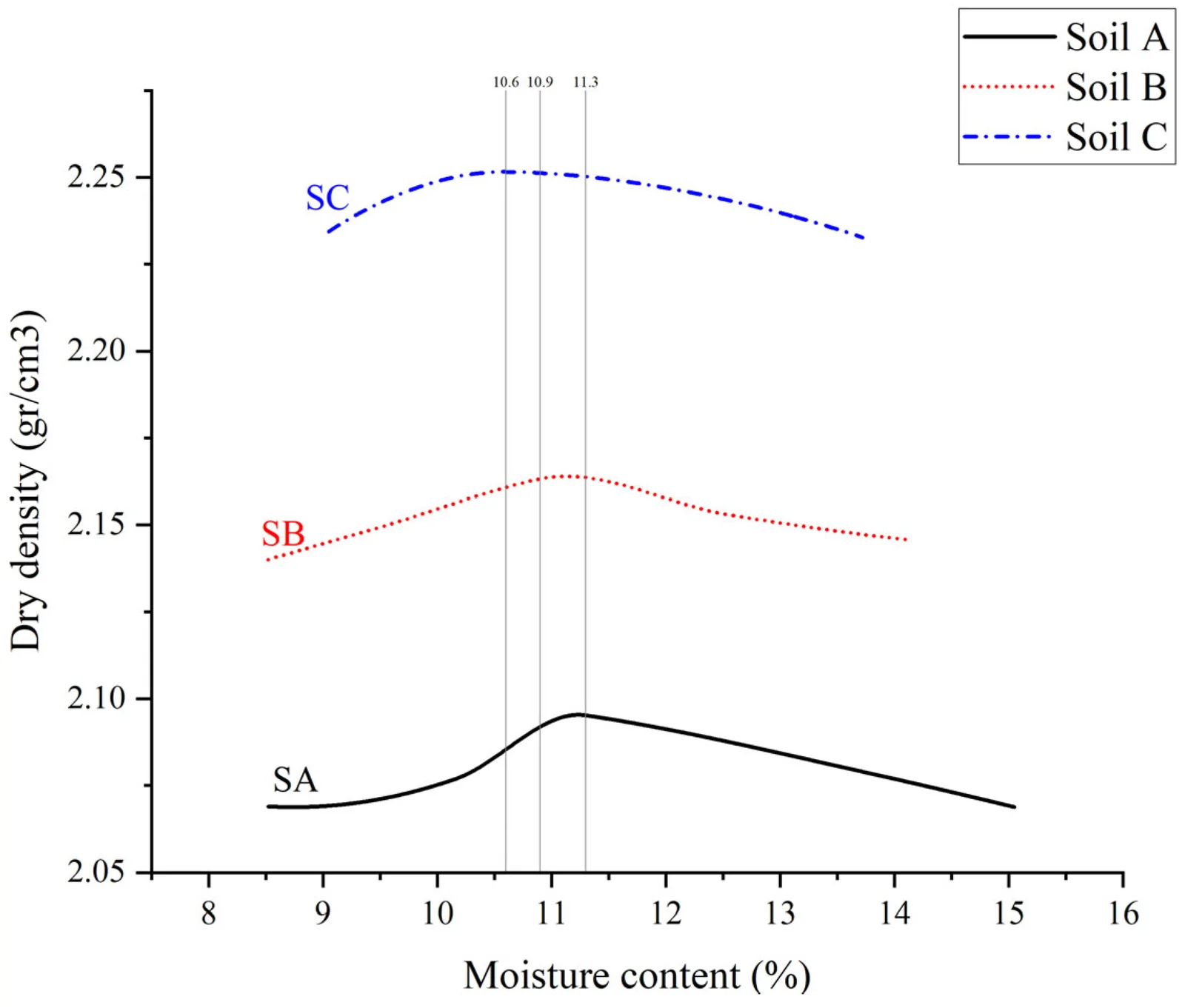
Standard Proctor compaction curves and corresponding optimum moisture contents for SA, SB, and SC.

As shown in Figure 1, the OMC values obtained for SA, SB, and SC were 11.3%, 10.9%, and 10.6%, respectively. These values correspond to the maximum dry densities obtained from the respective compaction curves and are consistent with the values reported in Table 5.

### 3.2. Biopolymer Solution

Previous research suggests that moist mixing improves biopolymer–soil contact efficiency [16,34]. To prepare the chitosan solution, a 1% acetic acid solution was prepared by dissolving the required amount of glacial acetic acid in distilled water. The solution was then heated to 80 °C. Chitosan powder was gradually added to the solution, stirring constantly to ensure that it was completely dissolved.

The crosslinked soy protein–PVA solution was prepared by first dissolving 5 g of soy protein and 5 g of PVA separately in 100 mL of deionized water at 60 °C under continuous stirring for 30 min. Once the solutions were homogeneous, oxidized glucose (glucose dialdehyde) was prepared by treating 2 g of glucose with 0.2 g of sodium periodate in 50 mL of deionized water for 1 h. The oxidized glucose solution was then added to the soy protein–PVA mixture, maintaining a 1:1 ratio between the soy protein–PVA mixture and the oxidized glucose solution. The mixture was stirred at 60 °C for 15 min to promote crosslinking between the soy protein and PVA. Afterward, the solution was allowed to cure at 60 °C for 24 h to form a crosslinked network, enhancing its strength and stability. The final solution was then cooled to room temperature and stored for further applications. All experiments were conducted at room temperature (23 ± 2 °C) unless otherwise specified.

### 3.3. Treated Soil Preparation

**Figure 2:**
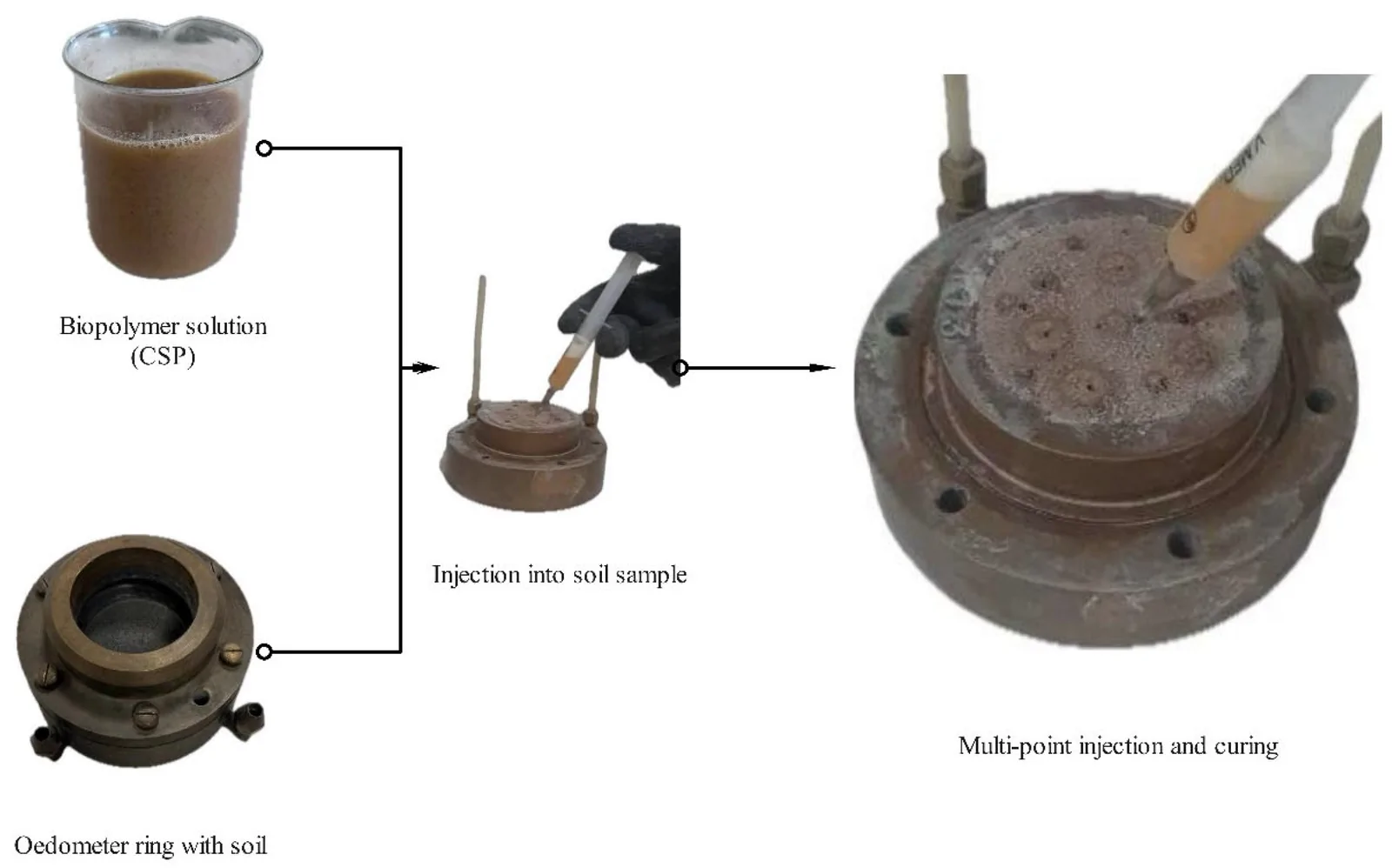
Sample preparation of CSP-treated collapsible soil for collapsible potential test.

Haeri (2014) indicated that the collapse potential is influenced by sample disturbance [1]. Therefore, to accurately evaluate the impact of the biopolymer, techniques that lead to sample disturbance must be avoided, as distinguishing between the effects of disturbance and improvement becomes difficult. In this study, a soil improvement method using injection was employed to minimize sample disturbance. As shown in Figure 2, the entire solution was injected into various points of the soil sample using a syringe. The injection points were selected to ensure uniform distribution of the solution throughout the sample. To further improve distribution uniformity, the biopolymer solution was injected gradually in multiple stages at several predefined locations throughout the specimen volume. After injection, the specimens were allowed to cure for a sufficient period to allow the solution to redistribute within the soil matrix. Although no direct quantitative assessment of biopolymer distribution was performed, visual inspection during specimen preparation indicated relatively uniform infiltration throughout the sample.

After the curing period, the sample was placed in an oedometer and subjected to loading up to a stress of 200 kPa at its natural moisture content. The sample was then saturated, and the settlement was measured over 24 h. The collapse potential was calculated using Equation 1. Although controlled injection and mixing procedures were used to improve uniformity, minor local heterogeneity in biopolymer distribution may still exist within treated specimens. All specimens were cured until nearly dry before testing so that the test conditions would be comparable. Specimens were also tested for residual moisture content, which was less than 1.5%, thereby reducing the effect of variation in moisture on the measured engineering properties.

### 3.4. Collapse Test

To assess the soil collapsibility potential, the ASTM D5333 standard was employed [35]. In the first stage, an initial stress of 5 kPa was applied. The samples were subsequently subjected to sequential stress levels of 12.5, 25, 50, 100, 200, and 400 kPa, with settlement measurements recorded at hourly intervals. Once the stress reached 200 kPa, the samples were submerged in water for one hour to achieve saturation. Loading was then continued under saturated conditions for up to 24 h, after which the applied stress was increased to 400 kPa. The final settlement of the samples under these conditions was measured.

The collapsibility potential (CP) was calculated as the ratio of the change in sample height to its initial height. The formula proposed by Jennings and Knight (1975) was employed to determine CP [36]:

Equation (1): Collapse potential

(1) $$CP=∆e/(1+e_{0})=(∆H/H_{0})$$

The collapse potential (CP) is used to quantify the likelihood of collapse settlement in a soil layer at a specific location. The settlement of a soil layer under applied vertical stress is determined by multiplying CP by H/100, where H is the thickness of the soil layer [35]. Table 6 Shows the ranges for the degree of collapse. 

### 3.5. Permeability Testing Preparation

This section focuses on the permeability test. Three types of soil with different clay contents (A, B, and C) were selected, and the biopolymers chitosan (CH) and CSP were injected at concentrations of 0.25%, 0.5%, and 1%. To assess the impact of biopolymer injection on soil permeability, a constant-head permeability test was conducted following ASTM D2434 on the samples after 9 days of curing [37].

The soil samples were placed in the permeability cell after being dried. To ensure uniform distribution, the biopolymers were dissolved in water and injected into the samples under carefully controlled pressure.

A modified constant-head procedure based on the principles of ASTM D2434 was used. A constant hydraulic head was maintained while the volume of discharged water was recorded at regular intervals. For specimens exhibiting very low flow rates, monitoring was continued over extended collection periods until a stable flow condition was obtained. The same apparatus and procedure were used for all specimens to ensure consistent comparisons, and hydraulic conductivity was calculated using Darcy’s law.

### 3.6. Unconfined Compressive Strength Test (UCS)

A customized mold was constructed to ensure compact and uniform specimens in compliance with ASTM D4609 and ASTM D2850 [38,39]. The mold had an inner diameter of 50 mm and a height of 200 mm. The 200-mm height refers to the mold height rather than the final specimen height. The mold was intentionally designed with an excess height to prevent soil loss during filling and static compaction. After compaction and extrusion, the final UCS specimens had a diameter of 50 mm and a height of approximately 100–110 mm, corresponding to an H/D ratio of approximately 2.0–2.2. The inner sides of the mold, hammer, and plug were slightly lubricated to facilitate specimen extrusion. Standard Proctor compaction tests were conducted on SA, SB, and SC to determine the optimum moisture content (OMC) and maximum dry density (MDD). The OMC values obtained from the original compaction data were 11.3%, 10.9%, and 10.6% for SA, SB, and SC, respectively. The corresponding compaction curves are presented in Figure 1, and the compaction parameters are summarized in Table 5. The effect of the biopolymer on the mechanical behavior of the treated specimens was assessed by adding it at different concentrations of 0, 0.25, 0.5, 1, and 1.5% by dry weight of soil.

It is important to note that the dry densities obtained for SA and SB were very close to their respective MDD values at their OMC.

The prepared biopolymer–soil mixture was then carefully poured into the mold in one layer. A hydraulic jack was used to statically compact the sample to reach its target density. Subsequently, the compacted sample was extracted by the hydraulic jack and cured in a controlled chamber with a temperature of 23 °C and 50–60% relative humidity.

The compressive strength test was conducted using a Universal Testing Machine (UTM; Instron 34TM 10) according to ASTM D2166 [40]. The axial displacement rate was set at 1 mm/min, corresponding to approximately 0.91–1.00% axial strain per minute for the final specimen height of 100–110 mm. Three specimens were tested for each condition, and the average value was reported.

### 3.7. Static Triaxial Testing Preparation

Triaxial tests were performed only on Soil C samples because this soil contained the highest clay fraction among the tested soils and was considered the most critical condition for evaluating the effectiveness of CSP stabilization, with specimens tested in the untreated condition and after treatment with chitosan (CH) and CSP at biopolymer contents of 0.25%, 0.5%, and 1%. Samples were cured for 9 days under controlled conditions (23 °C and 50–60% relative humidity), similar to those used for the UCS samples. The tests were carried out using a consolidated drained (CD) procedure according to ASTM D7181 [41]. Samples were enclosed in rubber membranes, and porous stones were placed at the top and bottom of the samples. The setup was connected to a computer-controlled system with an external LVDT mounted on the chamber cap for axial strain measurement. The strain rate was set at 0.1 mm/min, and confining pressures of 50, 100, and 200 kPa were applied.

### 3.8. SEM Observations

SEM imaging was conducted to examine the microstructure of untreated and treated samples. To ensure dryness, the samples were kept in the oven at 35 °C for 24 h. Then, carbon conductive connectors were used for attachment to the SEM mount. Samples were then coated with conductive tape after sufficient electrical grounding was achieved through carbon paint. A Zeiss Sigma VP field emission scanning electron microscope (Oberkochen, Germany) was used to observe the specimens.

### 3.9. FTIR

FTIR is a widely used technique for analyzing the chemical composition of materials. Within the 400–4000 cm^−1^ wavenumber range, spectral analysis was performed with Bruker Tensor 27 and CARY 630 spectrometers (Ettlingen, Germany). Additionally, to identify the characteristic bands of the biopolymer and soils, FTIR analysis was performed using a PerkinElmer System Series 2000 spectrophotometer (Shelton, CT, USA) over the same wavenumber range, with a resolution of 4 cm^−1^ and a scan speed of 0.5 cm/s. Samples were prepared using the potassium bromide (KBr) disc technique at a 100:1 (KBr to soil) ratio to provide clearer spectra. Table 7 summarizes the experiments performed in this study.

## 4. Results

### 4.1. Microstructural and Molecular Interactions in Biopolymer-Treated Soil Composites

This section discusses interactions involved in the formation of soil–biopolymer composites. The properties of biopolymer-treated soil are characterized using SEM and FTIR. SEM provides high-resolution imaging that enables detailed visualization of the composite microstructure, providing useful insights into the biopolymer–soil interactions. FTIR, on the other hand, provides information on the molecular composition of the materials and their interactions.

#### 4.1.1. SEM Images

**Figure 3:**
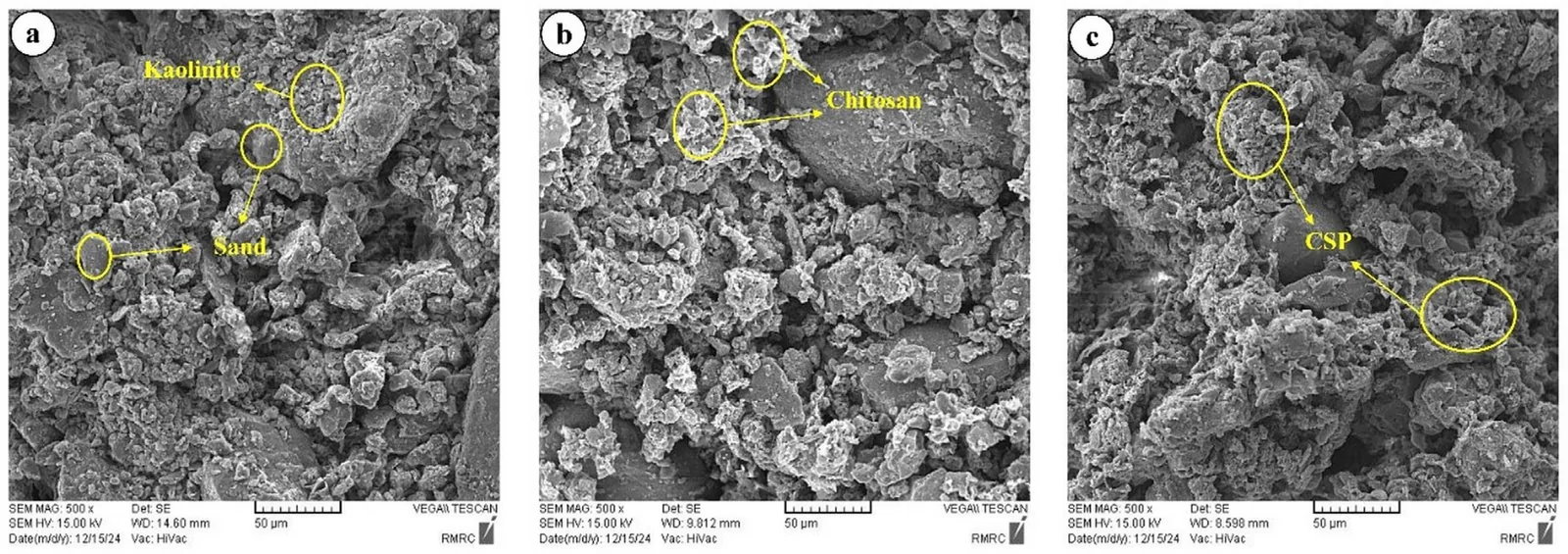
SEM images for untreated and treated samples: (**a**) Kaolinite (SC), (**b**) SC-CH, (**c**) SC-CSP.

Figure 3 presents the SEM images of SC, SC-CH, and SC-CSP samples. The sand particles exhibit a relatively angular and granular morphology, while kaolinite appears as irregular flakes attached to the sand grains. Compared with the untreated sample, the SC-CH specimen shows a more compact microstructure with reduced visible pore spaces. The SC-CSP sample exhibits a denser appearance and enhanced particle bonding, which qualitatively suggests the formation of a more interconnected structure due to biopolymer addition.

These observations are consistent with the measured improvements in mechanical strength and reductions in permeability; however, the SEM images provide qualitative evidence of microstructural changes rather than direct quantitative measurements of porosity.

It should be noted that SEM observations require drying and conductive sample preparation; therefore, the obtained images represent the dried state of the specimens rather than their in situ saturated condition.

#### 4.1.2. FTIR Spectra

**Figure 4:**
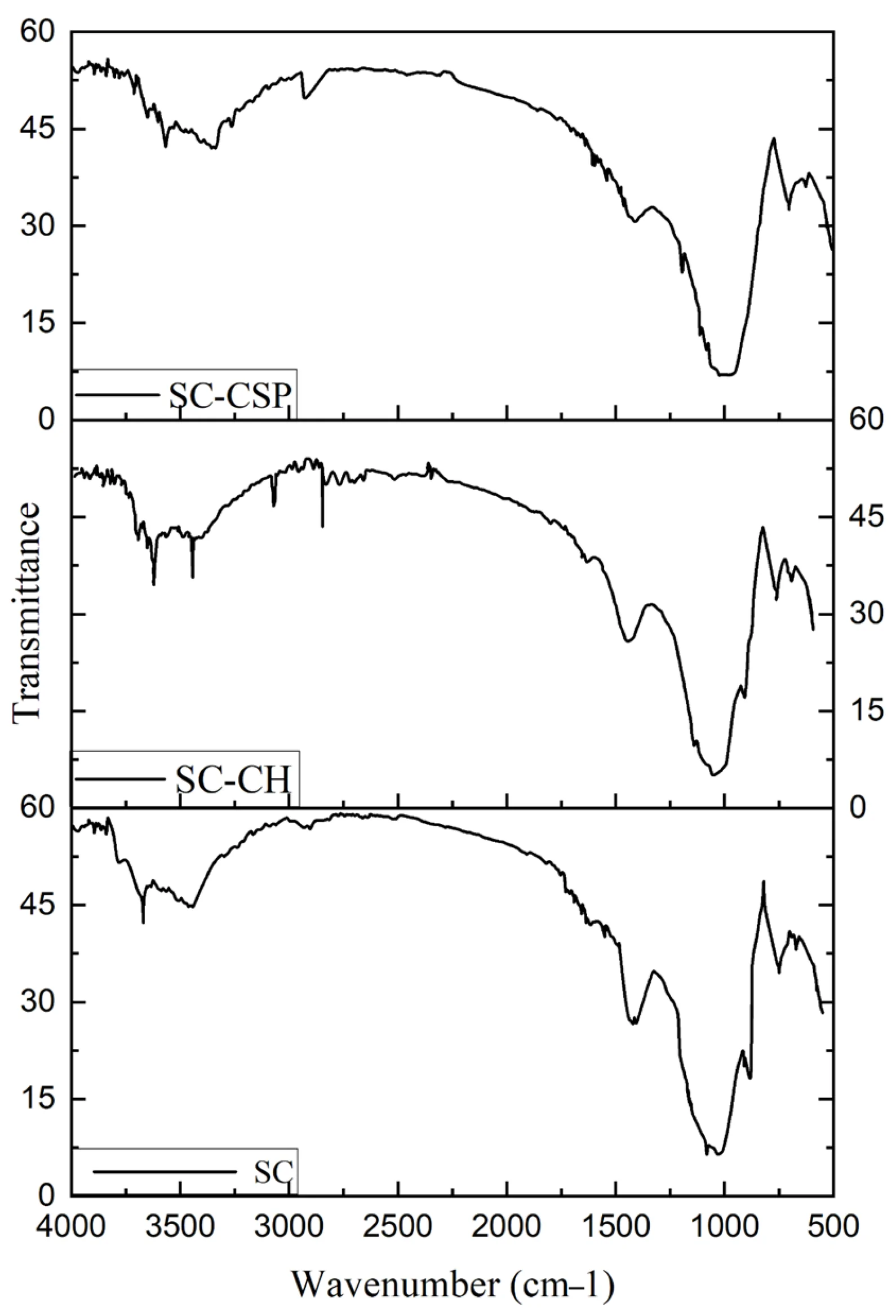
FTIR spectra of untreated soil and biopolymer-treated soils.

Figure 4 shows the FTIR spectra of SC, SC-CH, and SC-CSP samples. In the present study, FTIR analysis was used to identify spectral features associated with the soil and CSP constituents and to assess possible interactions among them. However, because several bands may arise from overlapping contributions of the individual components, specific crosslinking-related interactions cannot be assigned with complete confidence. Accordingly, the FTIR results provide qualitative and supportive evidence of changes in the composite system but cannot, by themselves, establish covalent crosslinking.

The SC spectrum indicates characteristic absorption bands at 3700 and 3650 cm^−1^, attributed to OH stretching vibrations. The band at 3620 cm^−1^ corresponds to the inner hydroxyl group (Al-OH) in the octahedral sheet of kaolinite. Also, the band at 3445 cm^−1^ corresponds to OH stretching, while the bands at 1631 cm^−1^ and 1471 cm^−1^ are associated with the H-O-H bending vibrations of water molecules adsorbed on the sample surface. The strong absorption bands at 1110, 1052, and 1010 cm^−1^ are attributed to Si-O stretching in kaolinite and quartz. The band at 905 cm^−1^ is attributed to Si-OH vibrations, while the band at 758 cm^−1^ corresponds to Si-O-Al vibrations.

Additionally, the weak absorption bands around 600 cm^−1^ are associated with Si-O-Si bending vibrations [42,43,44]. The FTIR spectrum of SC-CH exhibits a characteristic broad band centered at 3445 cm^−1^, attributed to the overlapping stretching vibrations of N-H and OH groups. The broad and weak bands around 3000 cm^−1^ are related to the C-H vibration modes of the chitosan polymer.

The weak bands observed around 2800–3000 cm^−1^ in the FTIR spectrum of SC-CSP are consistent with C–H stretching vibrations associated with organic components. The spectrum also exhibits a stronger absorption band at 3260 cm^−1^, likely due to the presence of free and bound OH groups in PVA [45,46]. The stretching vibrational bands between 1600 and 1700 cm^−1^ are attributed to the C=O groups in soy protein and PVA. Other bands in the FTIR spectrum of the SC-CSP sample may reflect overlapping contributions from PVA, oxidized glucose, and soy protein. The absorption bands appearing between 1000 and 1200 cm^−1^ may include overlapping contributions from organic C–O vibrations and soil Si–O stretching [47,48].

The FTIR spectrum of the SC-CSP sample contains bands consistent with contributions from soil, PVA, oxidized glucose, and soy protein; however, overlapping bands limit the unambiguous assignment of individual contributions. Identifying the precise interactions between PVA, soy protein, and oxidized glucose can be challenging due to the complexity of the crosslinking process and the potential for various chemical reactions. However, given the nature of their functional groups and the crosslinking process, two potential mechanisms can be considered: (1) the aldehyde groups generated during glucose oxidation could potentially react with amino groups in soy protein and hydroxyl groups in PVA; (2) strong hydrogen bonding may occur between their polar functional groups, enhancing the overall stability and cohesion of the mixture. The observed bands around 1100–1000 cm^−1^ may include contributions from carbohydrate-related vibrations, although overlap with soil bands limits their specific attribution to oxidized glucose [49,50,51].

The FTIR spectra showed changes in band positions and intensities following treatment, consistent with possible interactions within the composite system. These spectral observations, together with the denser microstructure observed in SEM images, are consistent with the development of a more interconnected soil–polymer structure that may contribute to the improved engineering performance. However, they do not establish the covalent nature of the underlying interactions.

#### 4.1.3. Structural Interactions in the Composites

**Figure 5:**
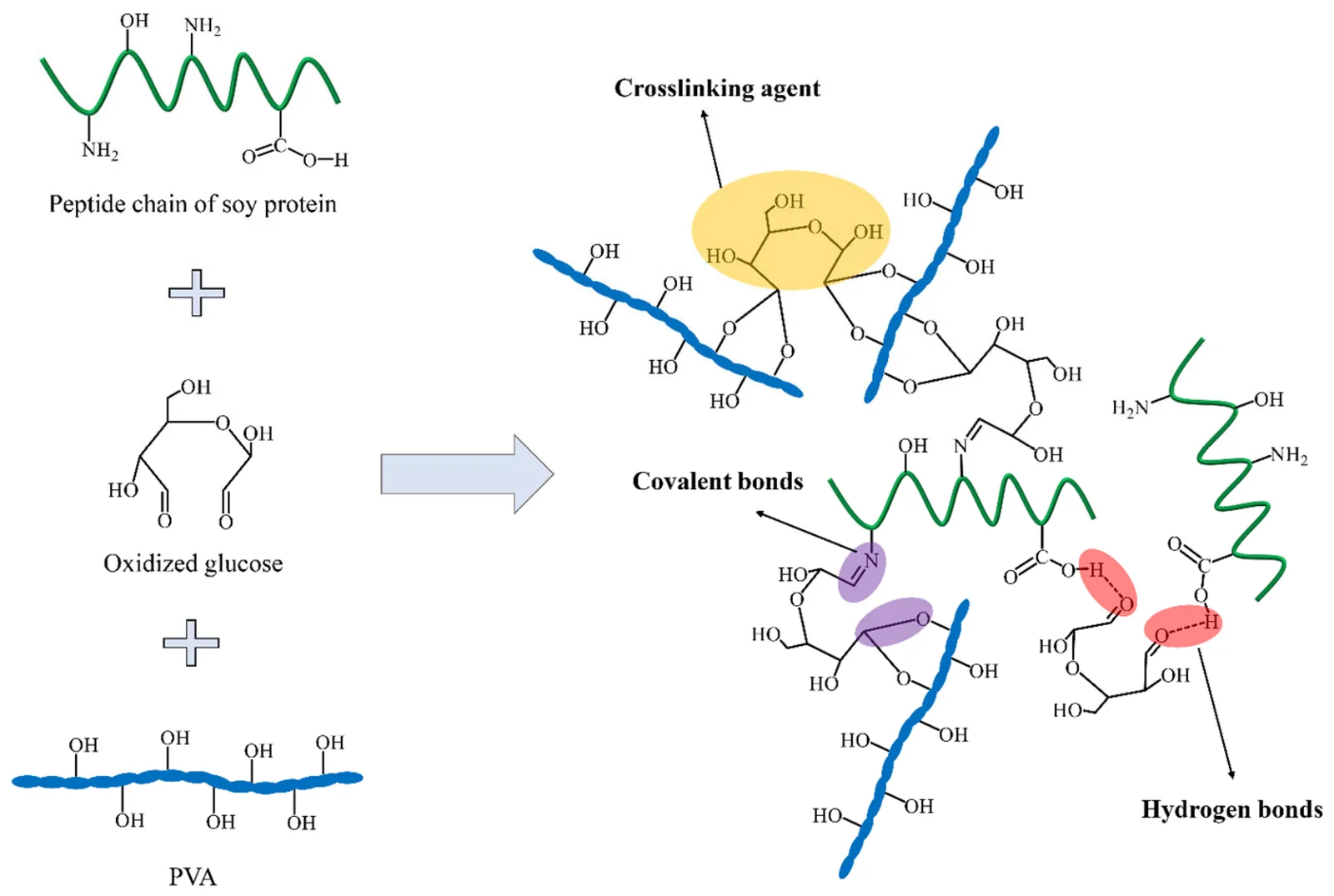
Schematic representation of the proposed interactions between PVA, soy protein, and oxidized glucose in the CSP-based binder.

**Figure 6:**
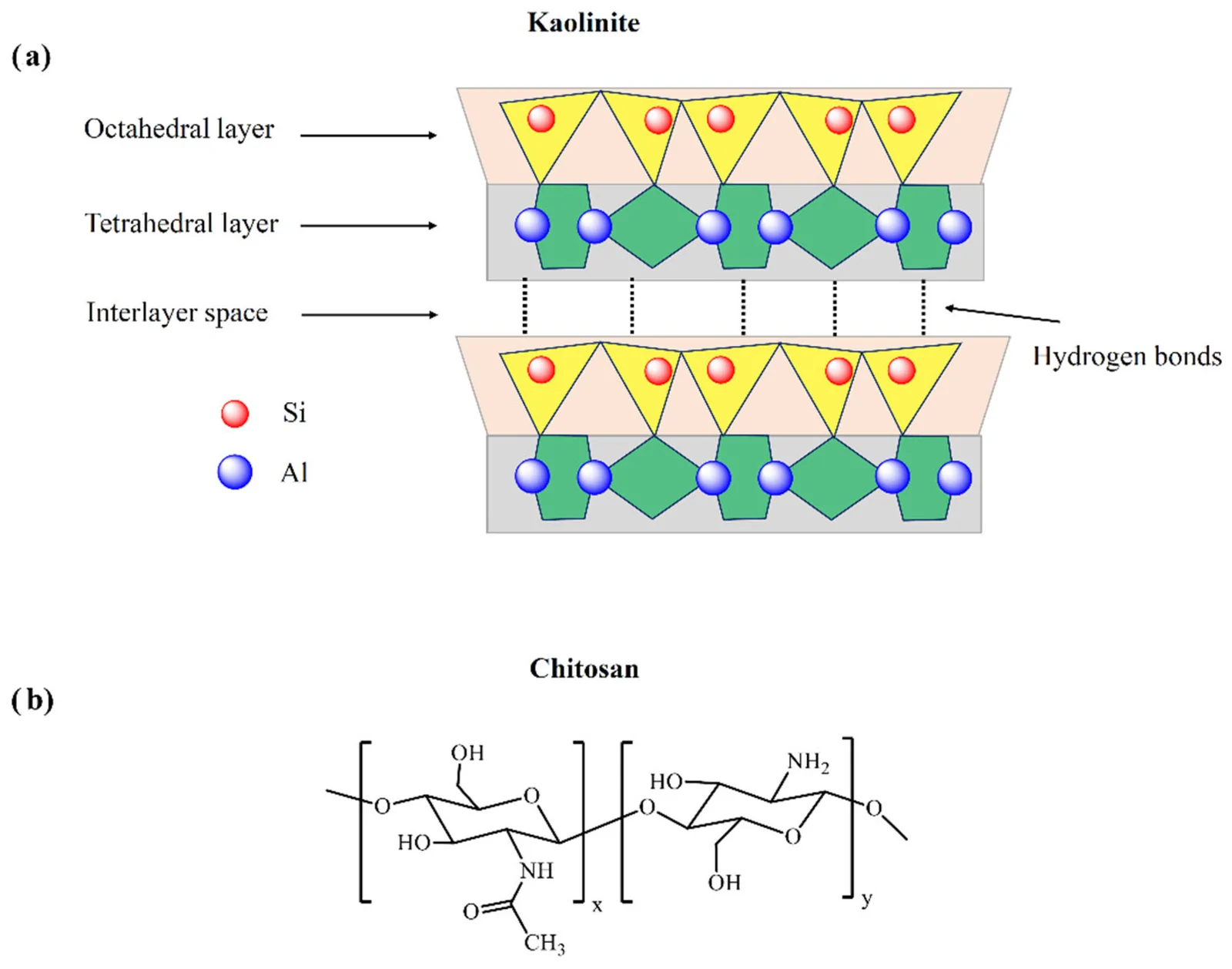
Schematic of kaolinite (**a**) and the chemical structure of chitosan polymer (**b**).

Kaolinite, a common dioctahedral 1:1 clay, is one of Earth’s most abundant phyllosilicates. Schematic illustration of the proposed molecular interactions among PVA, soy protein, and oxidized glucose in the CSP-based binder has been presented in Figure 5. The kaolinite structure comprises a tetrahedral silicon (Si) sheet sharing apical O^2−^ ions with an octahedral aluminum (Al) sheet (Figure 6a). Two types of charges, positive and negative, can be found on the kaolinite surface. The latter results from the protonation or deprotonation of hydroxyl groups at amphoteric sites, including the edges and octahedral faces [52,53,54].

Chitosan consists of N-acetylated glucosamine and glucosamine units, as shown in Figure 6. Chitosan is considered a weak base with low solubility in organic solvents. It is insoluble in neutral and alkaline environments, while it dissolves in dilute aqueous acidic solutions (pH < 6.5), where the glucosamine units are protonated, forming the soluble R-NH_3_^+^ species [55,56,57].

**Figure 7:**
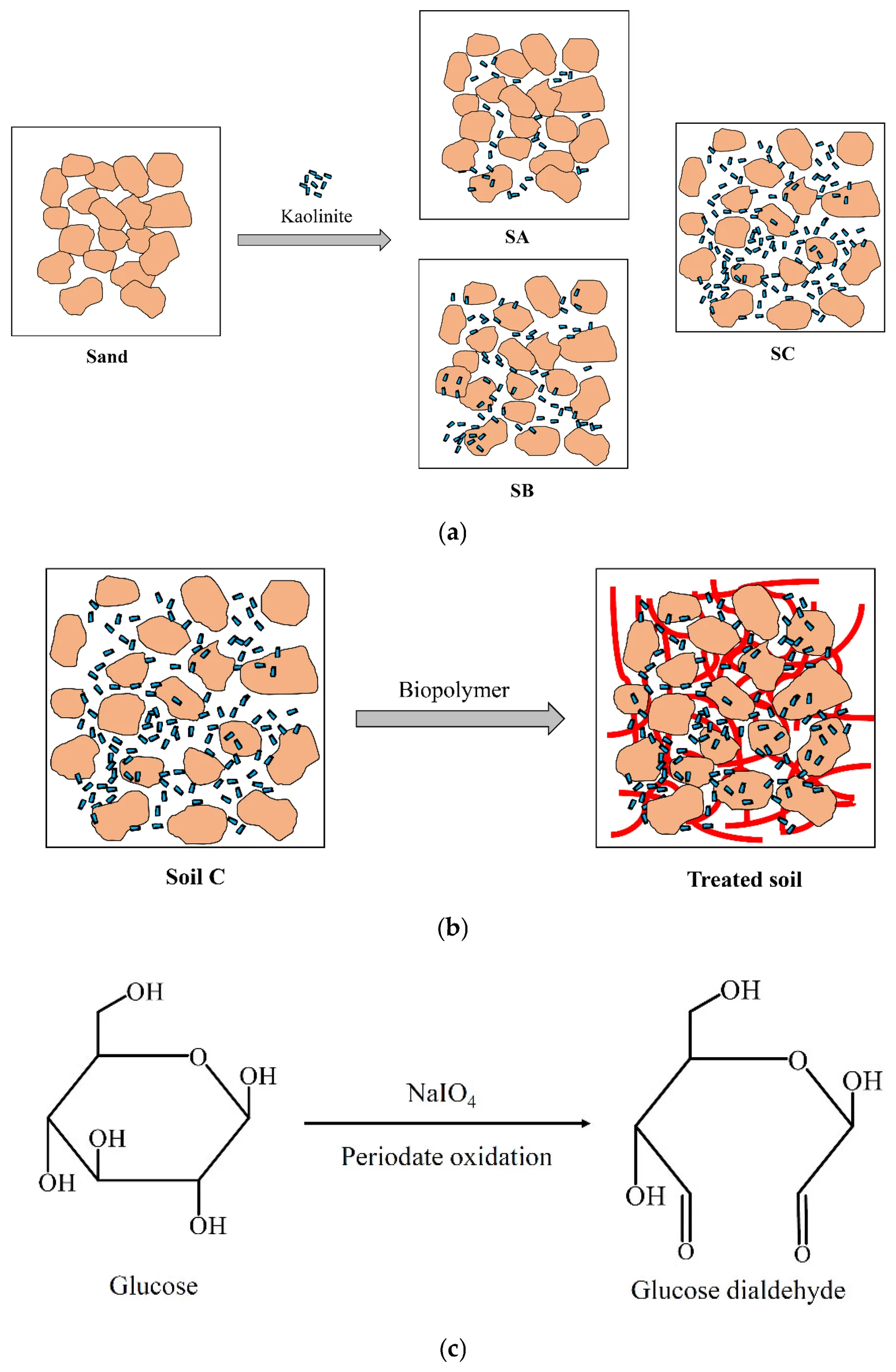
(**a**) Schematic of the kaolinite–sand mixture, (**b**) biopolymer-treated Soil C, (**c**) periodate oxidation of glucose.

Figure 7 presents a schematic representation of kaolinite–sand mixtures with kaolinite contents of 10% (SA), 15% (SB), and 20% (SC). Also, Figure 7b illustrates the schematic representation of the SC sample after biopolymer treatment. Incorporating CSP into the SC sample significantly enhances its mechanical properties. This enhancement is primarily due to the active functional groups within these biopolymers, which promote the formation and stabilization of the soil–biopolymer composite. CSP contains multiple active functional groups, including amine (-NH_2_), hydroxyl (-OH), and ketone (-C=O). These functional groups can form hydrogen bonds with the hydroxyl (-OH) sites on the kaolinite surface, strengthening the adhesion between the biopolymer and kaolinite particles.

Sand is mainly SiO_2_ and does not have sufficient functional groups to engage in significant hydrogen bonding with biopolymer molecules. Hence, the interaction between sand and the biopolymer network is mainly governed by two mechanisms: (1) interfacial mechanical interaction and (2) hydrophobic bonding. The interfacial mechanical interaction enhances the composite structure by creating polymer bonds around the sand grains. In addition, a hydrophobic interaction occurs at the interface between the polymer fibers containing nonpolar carbon chains and the sand grains with uncharged surfaces. In conclusion, incorporating chitosan and crosslinked soy protein–PVA into the kaolinite–sand mixture significantly enhances the composite’s mechanical performance. This improvement is primarily attributed to hydrogen bonding between biopolymers and kaolinite, along with interfacial mechanical and hydrophobic interactions within the sand–biopolymer system [58,59,60].

### 4.2. Collapse Potential Test

In this section, the effect of CSP on collapse potential (CP) is presented. CSP performance has also been compared with that of chitosan. The study evaluated three different collapsible soils with varying initial collapsibility, treated with two biopolymer-based stabilizers—CSP and chitosan. Each material was tested at three different concentrations (0.25%, 0.5%, and 1%), and the effects were analyzed over three curing durations (3, 6, and 9 days) to evaluate the evolution of soil stabilization over time.

#### 4.2.1. Stress–Strain Behavior

**Figure 8:**
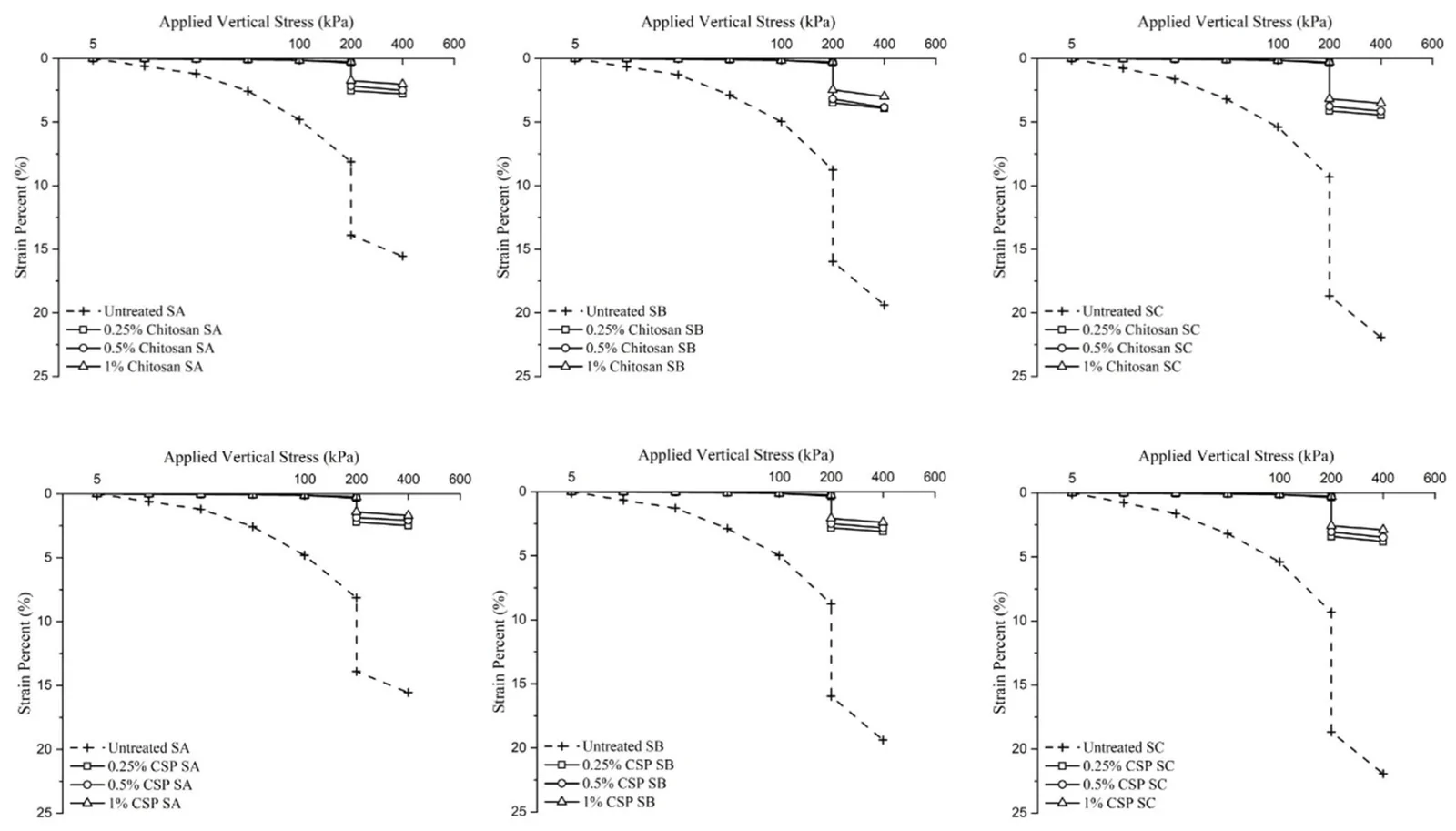
Stress–strain behavior of untreated and biopolymer-treated collapsible soils after nine days.

The stress–strain behavior of untreated and biopolymer-treated soils (after nine days) is presented in Figure 8. Three different soils were examined, as outlined in Table 5: Soil A (SA), Soil B (SB), and Soil C (SC). Regardless of the biopolymer type and content, the addition of biopolymers significantly reduced settlement, particularly under a load of 200 kPa. Additionally, a reduction in clay content resulted in a lower CP value. Soil A, which contains 10% clay, exhibited the lowest CP due to the presence of fewer compressible particles. With reduced clay content, the soil structure becomes more stable and less prone to collapse, thereby decreasing the overall collapsibility potential. This trend highlights the influence of clay fraction in determining the soil’s response to applied loads, reinforcing the role of particle distribution in controlling deformation.

Both CSP and chitosan effectively improved the CP of collapsible soils, with CSP-treated samples exhibiting lower settlements compared with those treated with chitosan. Furthermore, an increase in biopolymer content led to a greater reduction in settlement for both biopolymers. Among untreated soils, the highest axial strain under 200 kPa after 24 h was recorded for SC at 18.6%, whereas the lowest axial strain among treated soils was observed for 1%–CSP-SA at 1.4%. Additionally, a lower clay content correlated with reduced settlement. A comparative analysis of CSP and chitosan treatments indicates that CSP resulted in lower settlements, likely due to its ability to form a more robust network within the soil structure. This enhanced network reduces deformation and increases the soil’s load-bearing capacity. The observed improvement suggests that CSP is a promising stabilizing agent for collapsible soils, offering a viable alternative for enhancing soil performance in geotechnical applications.

#### 4.2.2. Factors Affecting the Reduction in Collapse Potential

**Figure 9:**
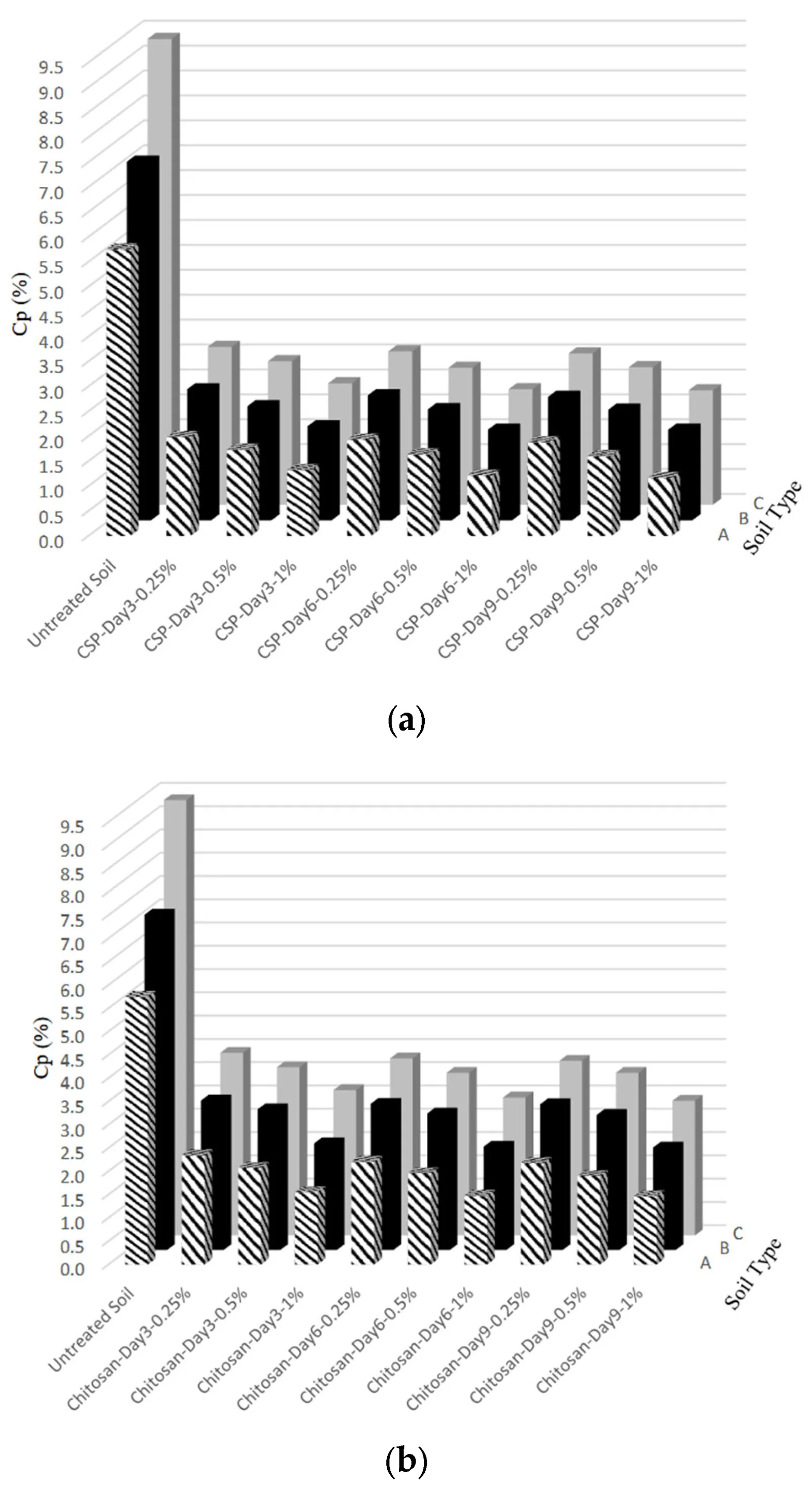
Effect of biopolymer content on the collapsibility potential (CP) of soils: (**a**) CSP treated soil, (**b**) chitosan treated soil

Biopolymer content plays a crucial role in reducing CP. Figure 9 presents the results obtained from the CP test, illustrating the effect of biopolymer content on soil collapsibility. The findings indicate that the concentration of biopolymeric additives has a substantial impact on CP reduction, with even small amounts leading to a significant decrease. For example, CP decreased from 9.3% for untreated Soil C to 3.0% for CSP-treated soil and 3.1% for chitosan-treated soil with just 0.25% biopolymer content. Furthermore, increasing the additive concentration from 0.25% to 1% consistently lowered CP across all soil samples (SA, SB, and SC), demonstrating the effectiveness of biopolymers in improving soil stability. Notably, compared to traditional stabilizers such as cement and lime, biopolymers can achieve a significant reduction in CP with considerably lower dosage requirements [61,62]. Therefore, biopolymers are considered an efficient and cost-effective alternative. Additionally, biopolymers do not introduce high alkalinity or adverse chemical reactions in the soil, which may help preserve its natural structure while improving mechanical properties.

In general, biopolymers enhance soil stabilization by forming a cohesive network within the soil matrix, improving structural integrity, and mitigating collapse potential (CP). CSP exhibited a more pronounced reduction in CP, likely due to its superior ability to create a denser and more stable polymer network. The enhanced bonding between CSP and soil particles further reinforces the structure, leading to reduced deformation under applied loads. A key factor in its effectiveness is also the crosslinking capability, which facilitates the formation of a more interconnected polymer network within the soil. This crosslinked network enhances load distribution, reduces water sensitivity, and improves the durability of treated soil under varying environmental conditions.

**Figure 10:**
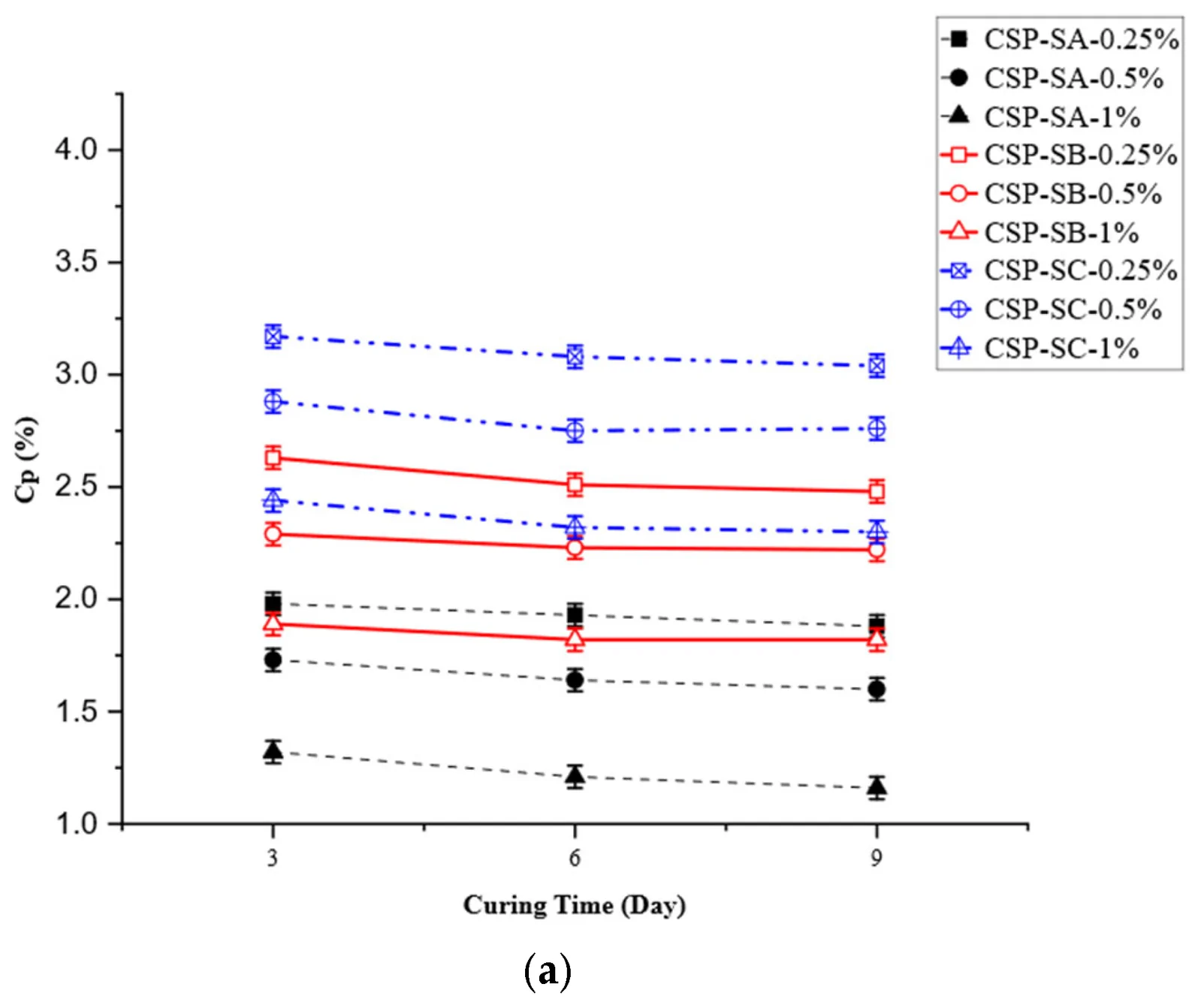

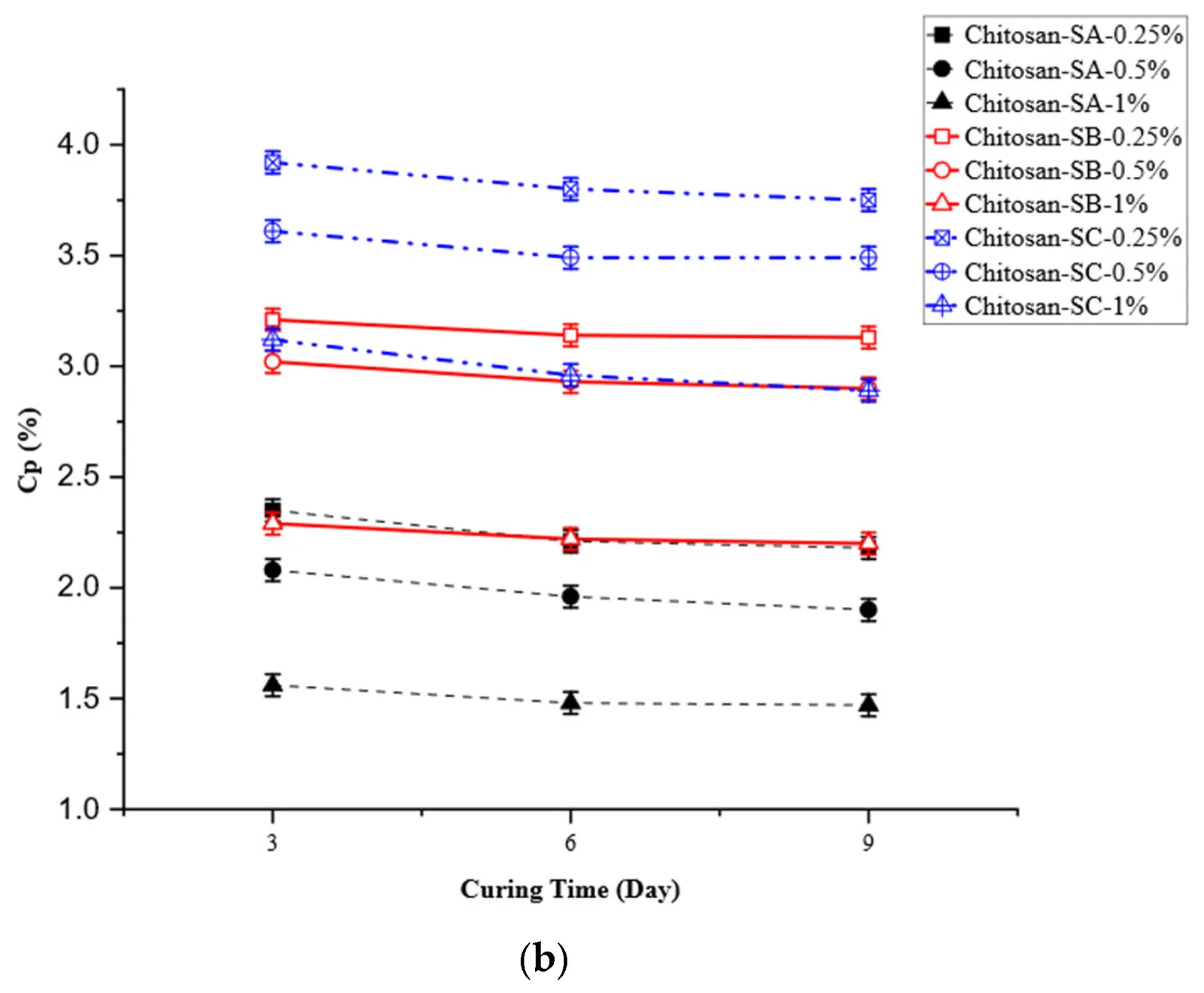
Effect of curing and dehydration time on CP variation in biopolymer-treated collapsible soil: (**a**) CSP treated soil, (**b**) chitosan treated soil

Another important factor influencing CP reduction is curing time and the associated variations in the moisture content of biopolymer-treated soil. Figure 10 shows the variation in CP after 3, 6, and 9 days for collapsible soils treated with CSP and chitosan. The overall trend indicates a gradual decrease in CP over time as moisture content decreases. As the treated soil loses moisture, the biopolymers may develop stronger intermolecular interactions and form a more interconnected network. This can increase soil cohesion and reduce its susceptibility to collapse. The graphs indicate that soils with higher clay content exhibit higher CP over time, regardless of biopolymer type. This is attributed to the greater compressibility and water retention of clay particles.

As shown in Figure 10, curing time has a notable effect on CP reduction, with values consistently decreasing from 3 to 9 days across all cases.

Both CSP and chitosan exhibited this trend, although CSP-treated soils showed a more pronounced reduction. The lowest CP value of 1.2% was obtained for CSP-SA-1% after 9 days of curing, emphasizing the combined influence of biopolymer content and curing duration on collapsible soil behavior. This suggests that prolonged curing allows for stronger soil–polymer interactions and contributes to improved soil stability.

### 4.3. Permeability

**Figure 11:**
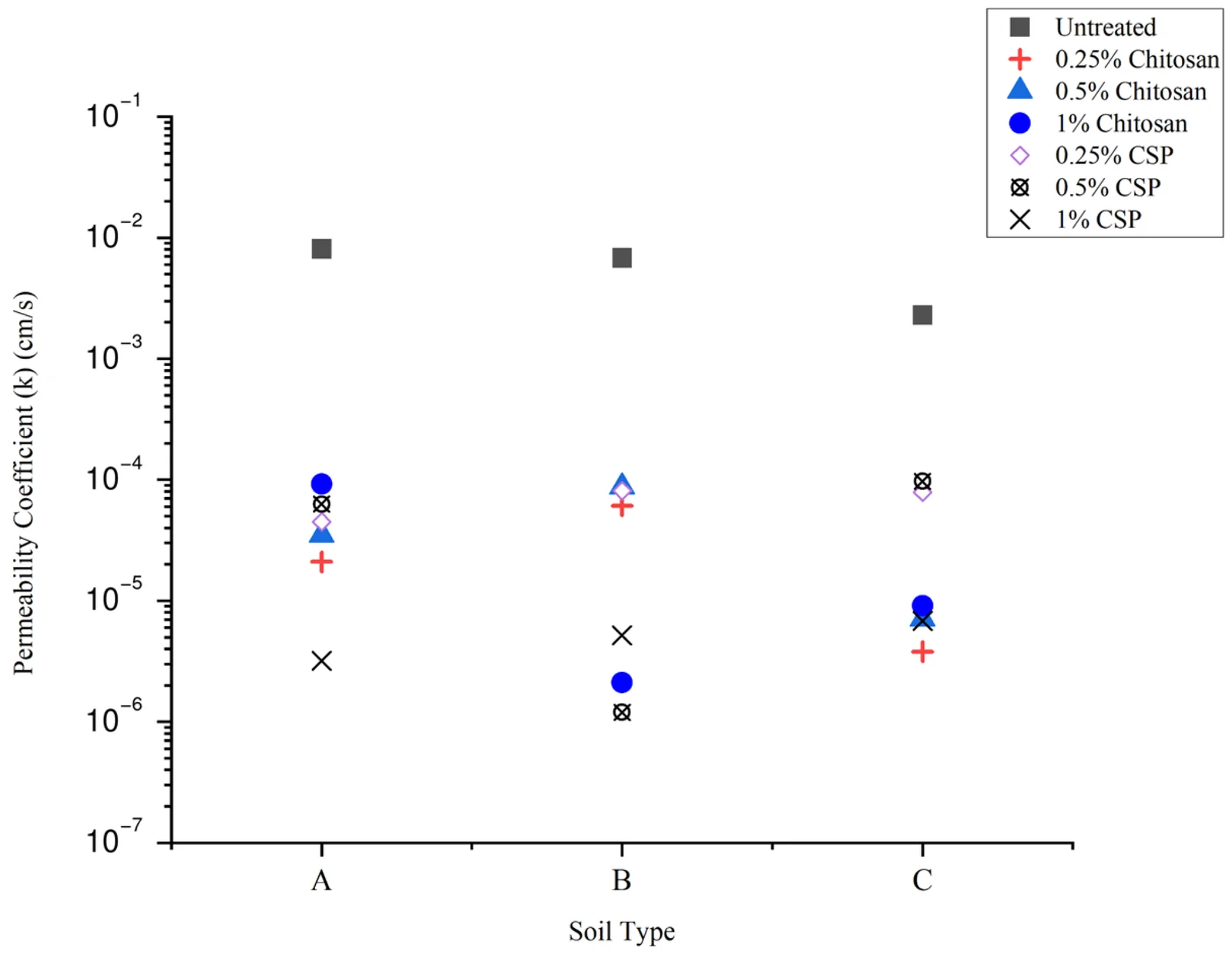
Permeability coefficient for different soil samples.

The permeability test results indicate a significant reduction in hydraulic conductivity for all soil types after biopolymer treatment, with CSP demonstrating the most substantial effect. Figure 11 presents the results obtained from the permeability test performed on samples after 9 days of curing. When Soil A and Soil B were untreated, their hydraulic conductivities were higher than that of Soil C. Their respective values were 8.1 × 10^−3^ and 6.8 × 10^−3^ cm/s, whereas the hydraulic conductivity of Soil C was 2.3 × 10^−3^ cm/s. However, upon stabilization, a substantial decrease was observed, particularly in CSP-treated samples. Its efficacy across various soil compositions is demonstrated by the fact that CSP, at a 1% concentration, decreased the hydraulic conductivity to 3.2 × 10^−6^ cm/s in Soil A, 5.2 × 10^−6^ cm/s in Soil B, and 6.8 × 10^−6^ cm/s in Soil C.

The reduction in hydraulic conductivity can be attributed to the ability of CSP to form an extensive polymer network within the soil matrix, which may partially fill or obstruct pore spaces and restrict water flow. The resulting network not only reinforces the soil but may also reduce water infiltration and limit moisture-induced degradation. This effect was particularly noticeable in soils that had a higher clay concentration (such as Soil C), which exhibited lower hydraulic conductivity than Soils A and B in the untreated condition. In contrast, chitosan treatment, while also effective, generally resulted in slightly higher hydraulic conductivity values compared with those obtained with CSP at the same concentration levels.

These findings underscore the potential of crosslinked soy protein–PVA (CSP) as a soil stabilizer, particularly in collapsible soils where reducing hydraulic conductivity is crucial for long-term stability. The effect of clay content on treatment effectiveness implies that different soil compositions may exhibit different responses to biopolymers, allowing for broader applicability in a range of geotechnical conditions.

### 4.4. UCS Test

**Figure 12:**
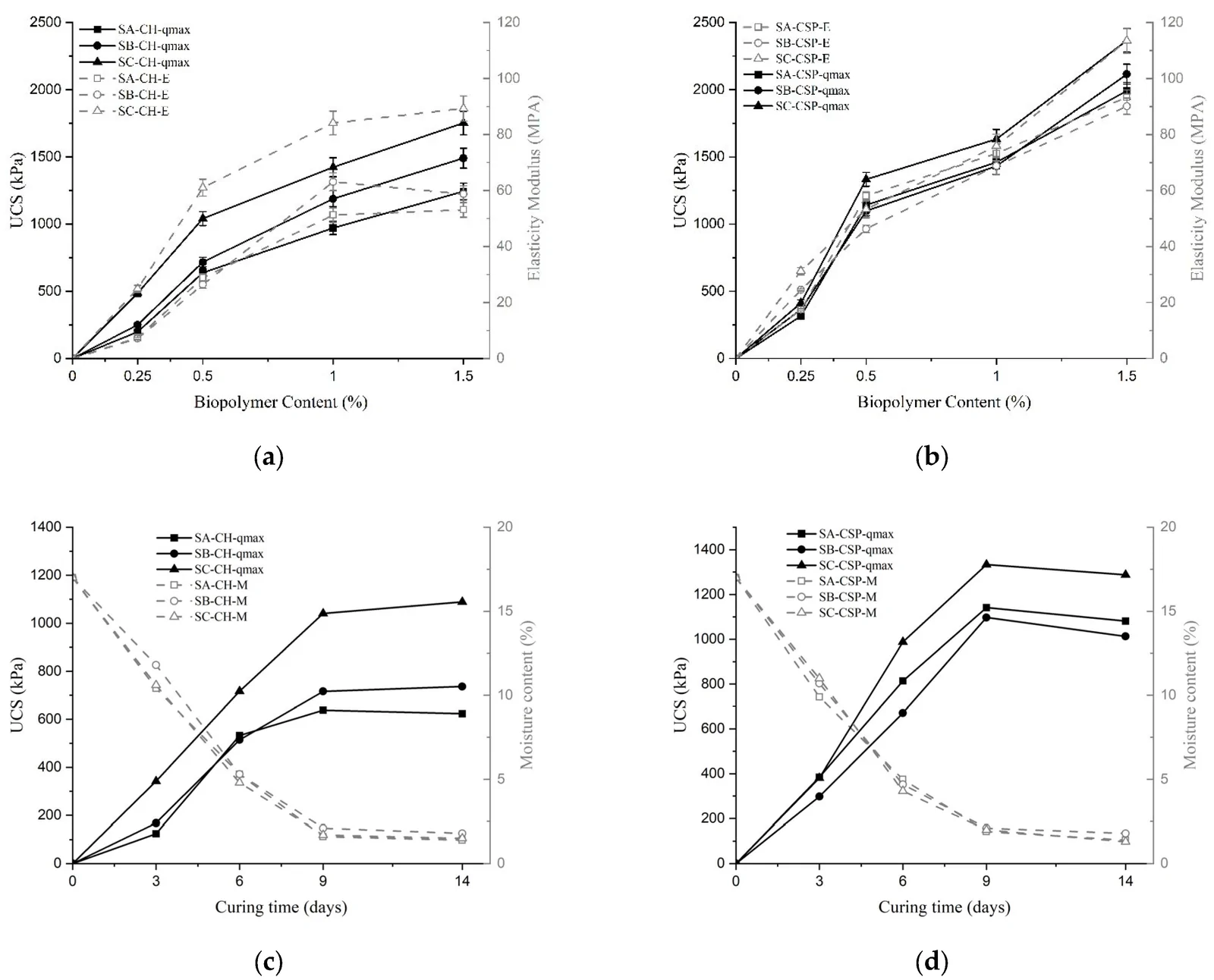
Unconfined compressive strength (UCS) and stiffness results: (**a**) biopolymer content effect for chitosan, (**b**) biopolymer content effect for CSP, (**c**) curing time effect for chitosan, (**d**) curing time effect for CSP.

The effect of biopolymer type, content, and curing time on the UCS of the three soil types was investigated. In all cases, adding biopolymers resulted in a substantial increase in compressive strength compared to that of untreated specimens. As expected, the untreated samples showed very low strength, and the specimens collapsed after being removed from the molds. Figure 12 presents the effect of biopolymer type (chitosan and CSP) and content on the UCS and elastic modulus (E) of the stabilized soil specimens. At 1.5% content and 9 days of curing, CSP-treated samples reached maximum UCS values of 1992 kPa, 2115 kPa, and 2367 kPa for SA, SB, and SC, respectively. On the other hand, the maximum UCS for chitosan-treated samples at the same concentration and curing duration was 1243 kPa (SA), 1490 kPa (SB), and 1752 kPa (SC). The CSP-treated specimens exhibited improved UCS and stiffness under the curing and testing conditions investigated, indicating enhanced mechanical performance compared with the untreated soil. The results also suggest improved resistance to the effects of the saturation conditions included in the experimental program. The elastic modulus (E) followed a similar trend to the UCS. For example, the E value of SC with 1.5% CSP reached 113.5 MPa after 9 days of curing, indicating a stiffer and more cohesive matrix. Among the soils with different clay contents and collapsibility potentials, SC exhibited higher UCS values compared to the other two soils, and SA exhibited the lowest strength. This may be attributed to the different amounts of fines content in each soil and their interaction mechanisms with biopolymers.

Curing time also had a pronounced effect, particularly during the initial stages. In general, moisture content decreased over time, and this reduction coincided with an increase in UCS. For instance, with 0.5% CSP in SC, UCS increased from 381.1 kPa at 3 days to 1333.0 kPa at 9 days. Similar trends were observed in other soils and for chitosan, though the magnitude of strength development was more significant in CSP-treated samples.

### 4.5. Static Triaxial Results

**Figure 13:**
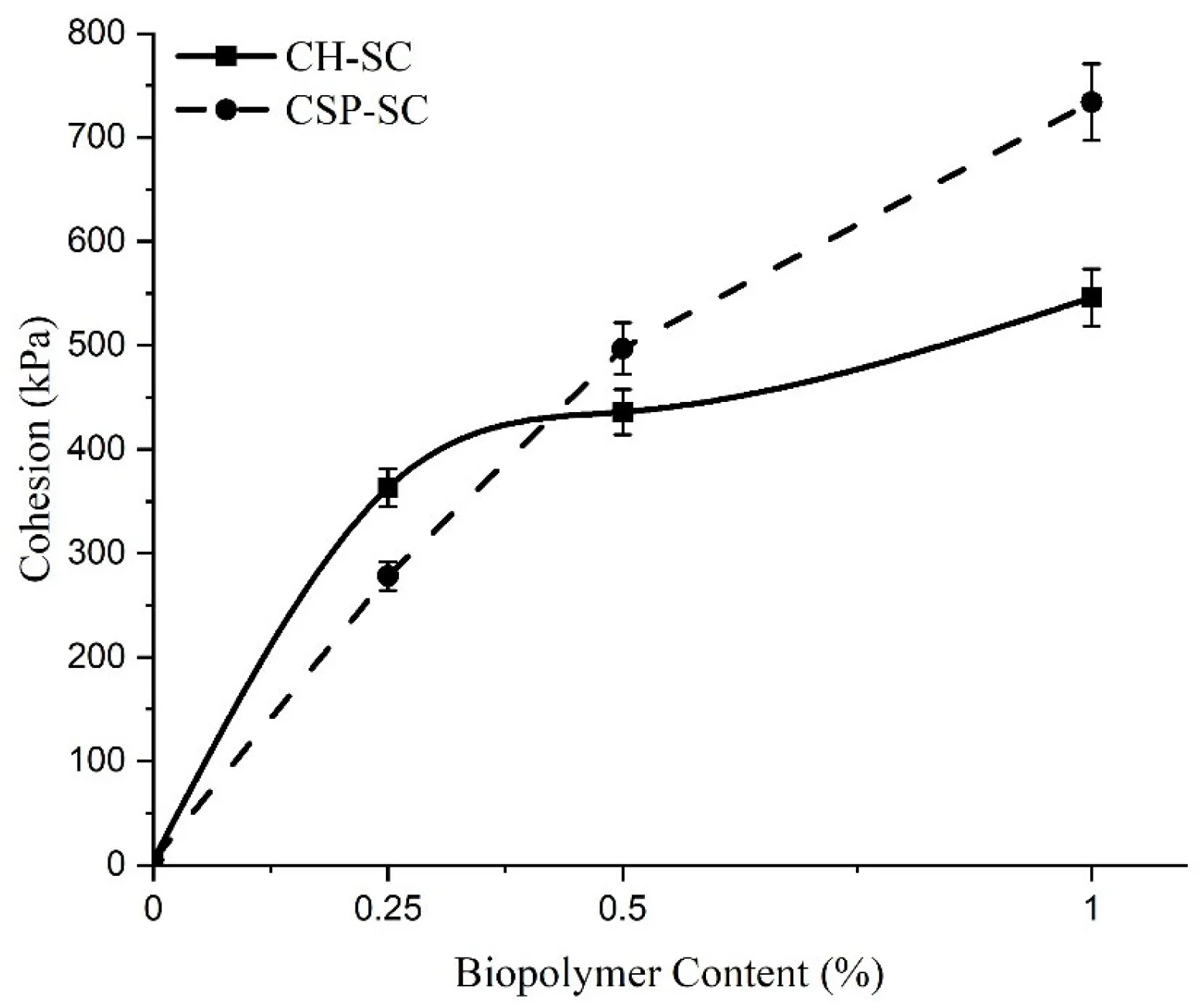
Cohesion variation for untreated and treated soils.

**Figure 14:**
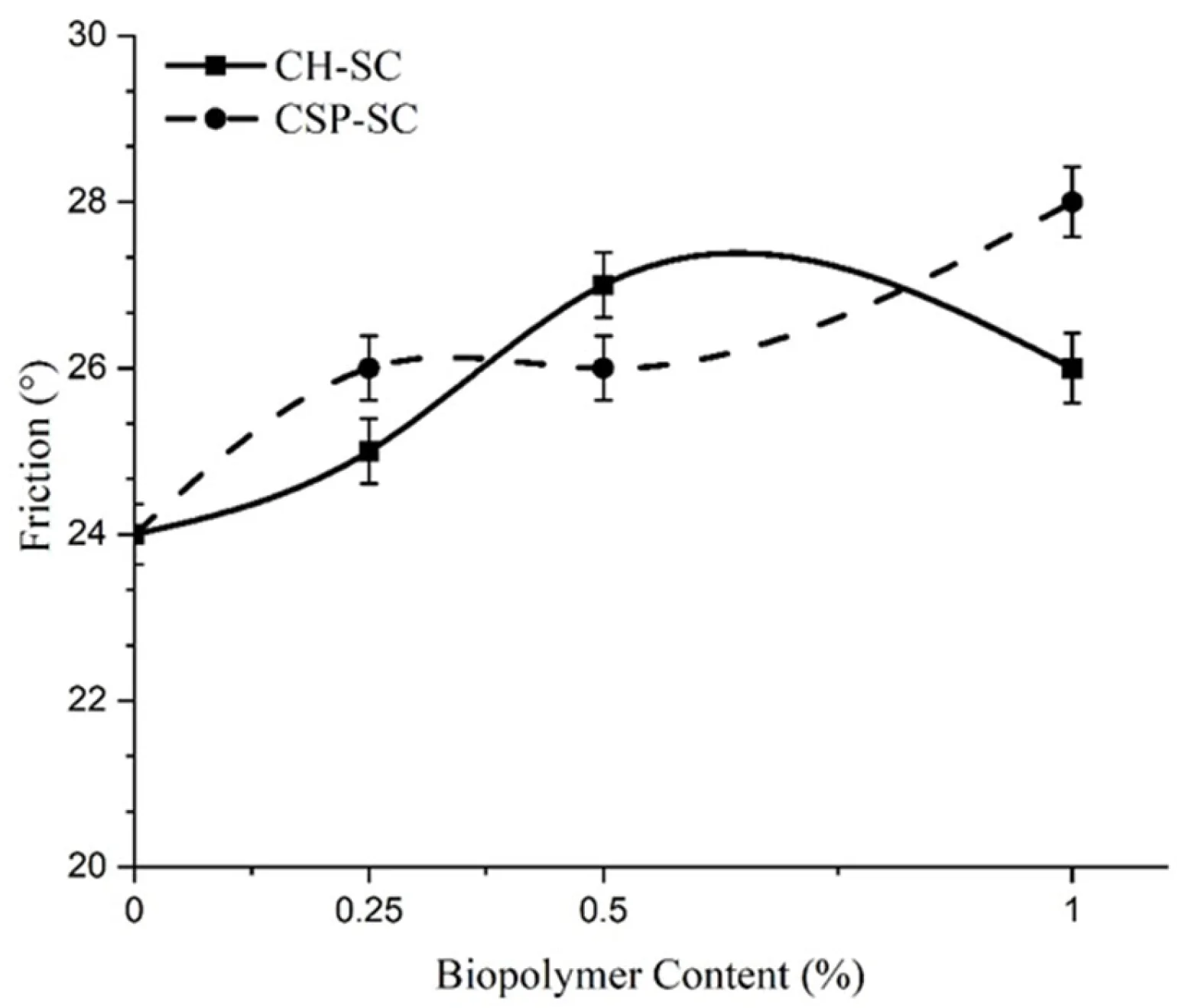
Internal friction angle variation for untreated and treated soils.

The results of the consolidated drained (CD) static triaxial tests can be seen in Figure 13 and Figure 14. In general, the addition of biopolymer significantly increased the shear strength of collapsible soil; however, the effect of the biopolymer was more pronounced in terms of cohesion. The untreated soil exhibited a cohesion of 5 kPa and an internal friction angle (φ′) of 24°. When CSP was added, cohesion increased to 287 kPa, 497 kPa, and 734 kPa at 0.25%, 0.5%, and 1% content, respectively. The friction angle (φ′) showed a much lower increase, reaching 28° at the highest biopolymer content. A similar trend was observed for the chitosan-treated Soil C samples, while the increase in cohesion was comparatively lower than that observed for CSP. Although CSP generally performed better at medium and high concentrations, chitosan-treated samples showed slightly higher cohesion at the lowest dosage level (0.25%). This behavior may be associated with the formation of initial interparticle interactions. However, as the biopolymer content increased, CSP may have developed a denser crosslinked network and achieved substantially higher shear strength under the tested conditions. At 0.25%, 0.5%, and 1% CH content, cohesion reached 363 kPa, 436 kPa, and 546 kPa, respectively.

These enhancements point to the formation of continuous polymer matrices that bind soil particles and may partially fill micro-voids, which leads to a denser and more cohesive structure. The dominant improvement in shear strength is attributed to the increase in cohesion rather than frictional resistance. This indicates that biopolymers act mainly by enhancing interparticle bonding. This may occur through hydrogen bonding, van der Waals forces, electrostatic interactions, and other interfacial interactions, depending on the polymer’s chemistry. The consistent rise in cohesion with increasing biopolymer content supports the effectiveness of biopolymer matrices in stabilizing collapsible soils, particularly in applications requiring enhanced shear resistance.

## 5. Discussion

### 5.1. Comparison with Previous Studies

This study’s findings align with previous research on biopolymer-based stabilization of collapsible soils. Ayeldeen et al. (2017) demonstrated that xanthan gum and guar gum could enhance soil strength and reduce collapse potential, but these benefits were primarily achieved at higher biopolymer concentrations [8]. Moreover, concerns remained regarding their sensitivity to moisture and long-term durability. Similarly, Habib et al. (2025) reported up to a 95% reduction in collapse potential using 4% sodium alginate; however, this was accompanied by a decline in maximum dry density and elastic modulus, indicating potential trade-offs in mechanical performance [30]. The same study also showed that biopolymers such as gelatin and sodium alginate can improve cohesion and reduce collapse susceptibility, but their effectiveness was often dosage-dependent and sometimes associated with reductions in the internal friction angle [30].

In the present study, CSP produced substantial improvements in compressive strength and cohesion while reducing collapse potential at the investigated concentrations. Compared with the concentrations reported for some previously studied biopolymers, these improvements were obtained at relatively low CSP contents. CSP-treated specimens exhibited improved mechanical performance under the saturation conditions included in the present experimental program. These results indicate that CSP treatment can maintain improved mechanical behavior during the specific saturation conditions investigated in this study. However, these observations should not be interpreted as evidence of long-term water resistance or durability under repeated or prolonged environmental exposure.

The present results also should not be interpreted as establishing long-term durability or fully resolving the moisture sensitivity and durability limitations reported for other biopolymer systems. The current study was conducted over relatively short curing periods and did not include systematic cyclic wetting–drying, long-term aging, or leaching experiments. Therefore, the observed performance provides an indication of the potential of CSP under the investigated conditions, while its long-term durability requires further experimental validation.

### 5.2. Limitations and Potential Field Applications

Despite the promising improvement in the geotechnical performance of CSP-treated collapsible soil, several limitations of the present study should be acknowledged. First, the investigation was conducted at laboratory scale using sand–kaolinite mixtures, and therefore the observed performance may not fully represent the heterogeneity and environmental variability encountered in field conditions. Second, although the mechanical and hydraulic responses were investigated under controlled curing conditions, the long-term durability of the CSP-treated soil under repeated wetting–drying cycles, temperature fluctuations, and biological activity was not systematically evaluated. Such environmental factors may influence the stability of polymer–soil interactions and, consequently, the long-term engineering performance of the treated soil. Third, the present study did not quantitatively evaluate the uniformity of CSP distribution during field-scale application, nor did it assess the practical requirements, cost, and scalability of the treatment. These aspects require further investigation through pilot-scale and field trials.

Nevertheless, the substantial improvements in collapse resistance, UCS, shear strength, and hydraulic behavior observed in this study indicate that CSP has potential for practical ground-improvement applications. Based on the enhanced geotechnical properties, potential applications include stabilization of collapsible soil layers, shallow slope and embankment surface protection, erosion-control measures, and stabilization of soils used in earthworks and infrastructure-related applications. In particular, the improvement in soil bonding and strength suggests potential for applications where reduction in collapse susceptibility and enhancement of near-surface stability are required. However, these applications should initially be considered potential uses rather than direct recommendations for unrestricted field implementation. Further field-scale studies are required to determine appropriate application methods, optimum treatment dosage, curing conditions, long-term durability, environmental compatibility, and economic feasibility under site-specific conditions.

## 6. Conclusions

This study investigated the use of crosslinked soy protein–PVA (CSP) and chitosan biopolymers to enhance the geotechnical performance of collapsible soils through a series of laboratory-scale experiments. Soils with varying clay contents were treated and evaluated using collapse potential and permeability tests, as well as microstructural analyses. The key findings of the study are summarized below:

- CSP generally outperformed chitosan, particularly at medium and high concentrations, by providing greater improvements in compressive strength and stiffness, as well as a reduction in collapse potential.
- The effectiveness of both biopolymers varied with clay content; however, CSP showed higher effectiveness in soils with 15–20% clay, which suggests stronger interactions with the fine-grained fraction.
- CSP-treated soils exhibited improved mechanical performance under the saturation conditions investigated, with the magnitude of improvement depending on clay content and CSP dosage.
- SEM observations showed a denser and more interconnected morphology in CSP-treated specimens compared with untreated soil.
- The FTIR results identified functional groups and spectral features consistent with interactions among the CSP constituents and the soil matrix.
- Hydraulic conductivity was also significantly reduced in CSP-treated soils under the investigated laboratory conditions, indicating restricted water flow compared with untreated soil.

The findings of this study are limited to laboratory-prepared sand–kaolinite mixtures and the curing periods investigated. Therefore, the present results do not establish the long-term durability, environmental safety, field-scale performance, or overall environmental superiority of CSP over conventional stabilizers. Further studies involving extended aging, repeated wetting–drying cycles, leaching and environmental assessment, field-scale validation, and life cycle assessment are required to evaluate the long-term performance, environmental implications, scalability, and practical applicability of the proposed binder.

Accordingly, the potential use of CSP in applications such as roads, railways, embankments, and building foundations should be considered a future research direction rather than a validated field application at this stage.

## Figures and Tables

**Table 1:** Mineralogical composition of sand.

Component	Percentage (%)
Quartz	76.5
Calcite	3.4
Dolomite	0.1
Siderite	0.7
Siderite (Mg/Ca)	2.2
Andradite	1.0
Plagioclase (An0-15)	7.7
K-Feldspar	3.0
Kaolinite	0.9
Chlorite	1.4
Illite	3.2

**Table 2:** Geotechnical properties of sand.

Soil Type	D50 (mm)	Cu	Cc	Gs	Shape	USCS	e_min_	e_max_
Sand	0.49	2.77	0.91	2.63	Round	SP	0.61	0.76

**Table 3:** Mineralogical composition of kaolinite.

Chemical Composition	Amount (%)
SiO_2_	72.39
Al_2_O_3_	18.84
Fe_2_O_3_	0.49
TiO_2_	0.16
CaO	0.2
MgO	0.11
Na_2_O	0.26
K_2_O	0.12
P_2_O_5_	<0.1
MnO	<0.1
S	0.2
Other Components	7.17

**Table 4:** Geotechnical properties of kaolinite [31,32].

Specification	Result	Standard
Specific Gravity	2.56	ASTM D 854-14
Plastic Limit (PL)	24.24%	ASTM D 4318
Liquid Limit (LL)	31.3%	ASTM D 4318
Classification (ASTM)	CL	—
pH	4.5	—

**Table 5:** Geotechnical properties of prepared collapsible soils.

Specimens	Soil Class	γ (kN/m^3^)	ω (%)	$\gamma_{d,max}$ (kN/m^3^)	ω*_opt_* (%)	Gs	Cu	Cc	LL	PL	PI	Clay (%)
Soil A (SA)	SW-SC	16.33	5	20.26	11.3	2.70	8.5	2.38	N/A	N.P.	N/A	10
Soil B (SB)	SW-SC	16.25	5	20.43	10.9	2.72	10.8	2.68	18.8	12.9	5.9	15
Soil C (SC)	SW-SC	16.08	5	20.97	10.6	2.73	12	3.85	24.3	14.5	9.8	20

**Table 6:** Classification of collapse potential (D5333-92).

Degree of Collapse	Collapse Potential (%)
None	0
Slight	0.1 to 2.0
Moderate	2.1 to 6.0
Moderately severe	6.1 to 10.0
Severe	>10

**Table 7:** Experimental program of this study.

Test Type	Biopolymer Type	Biopolymer Content (%)	Curing Time (days)	Soil Type
Collapse test	CSP, CH	0, 0.25, 0.5, 1	3, 6, 9	SA, SB, SC
Permeability	CSP, CH	0, 0.25, 0.5, 1	9	SA, SB, SC
UCS	CSP, CH	0, 0.25, 0.5, 1, 1.5	3, 6, 9, 14	SA, SB, SC
Static triaxial	CSP, CH	0, 0.25, 0.5, 1	9	SC
SEM	CSP, CH	0, 0.5	9	SC
FTIR	CSP, CH	0, 0.5	9	SC

## Data Availability

The original contributions presented in the study are included in the article; further inquiries can be directed to the corresponding authors.
